# The pan-plastome of tartary buckwheat (*fagopyrum tataricum*): key insights into genetic diversity and the history of lineage divergence

**DOI:** 10.1186/s12870-023-04218-7

**Published:** 2023-04-24

**Authors:** Jiawei Zhou, Wenchuang He, Jie Wang, Xuezhu Liao, Kunli Xiang, Mingchuan Ma, Zhang Liu, Yongyao Li, Luke R. Tembrock, Zhiqiang Wu, Longlong Liu

**Affiliations:** 1 College of Plant Science and Technology, Huazhong Agricultural University, Wuhan, 430070 China; 2 Shenzhen Branch, Guangdong Laboratory of Lingnan Modern Agriculture, Key Laboratory of Synthetic Biology, Ministry of Agriculture and Rural Affairs, Agricultural Genomics Institute at Shenzhen, Chinese Academy of Agricultural Sciences, Shenzhen, 518000 China; 3 College of Science, Health, Engineering and Education, Murdoch University, Western Australia, Perth, 6150 Australia; 4 State Key Laboratory of Systematic and Evolutionary Botany, Institute of Botany, Chinese Academy of Sciences, Beijing, 100093 China; 5 Center for Agricultural Genetic Resources Research, Shanxi Agricultural University, Taiyuan, 030031 China; 6 Shanxi Key Laboratory of Genetic Resources and Genetic Improvement of Minor Crops, Taiyuan, 030031 China; 7 Department of Agricultural Biology, Colorado State University, Fort Collins, CO 80523 USA; 8 College of Horticulture, Shanxi Agricultural University, Shanxi, 030801 China

**Keywords:** Tartary buckwheat, *Fagopyrum*, Pan-plastome, Population structure, Genetic diversity, Phylogeny

## Abstract

**Background:**

Tartary buckwheat (*Fagopyrum tataricum*) is an important food and medicine crop plant, which has been cultivated for 4000 years. A nuclear genome has been generated for this species, while an intraspecific pan-plastome has yet to be produced. As such a detailed understanding of the maternal genealogy of Tartary buckwheat has not been thoroughly investigated.

**Results:**

In this study, we *de novo* assembled 513 complete plastomes of *Fagopyrum* and compared with 8 complete plastomes of *Fagopyrum* downloaded from the NCBI database to construct a pan-plastome for *F. tartaricum* and resolve genomic variation. The complete plastomes of the 513 newly assembled *Fagopyrum* plastome sizes ranged from 159,253 bp to 159,576 bp with total GC contents ranged from 37.76 to 37.97%. These plastomes all maintained the typical quadripartite structure, consisting of a pair of inverted repeat regions (IRA and IRB) separated by a large single copy region (LSC) and a small single copy region (SSC). Although the structure and gene content of the *Fagopyrum* plastomes are conserved, numerous nucleotide variations were detected from which population structure could be resolved. The nucleotide variants were most abundant in the non-coding regions of the genome and of those the intergenic regions had the most. Mutational hotspots were primarily found in the LSC regions. The complete 521 *Fagopyrum* plastomes were divided into five genetic clusters, among which 509 Tartary buckwheat plastomes were divided into three genetic clusters (Ft-I/Ft-II/Ft-III). The genetic diversity in the Tartary buckwheat genetic clusters was the greatest in Ft-III, and the genetic distance between Ft-I and Ft-II was the largest. Based on the results of population structure and genetic diversity analysis, Ft-III was further subdivided into three subgroups Ft-IIIa, Ft-IIIb, and Ft-IIIc. Divergence time estimation indicated that the genera *Fagopyrum* and *Rheum* (rhubarb) shared a common ancestor about 48 million years ago (mya) and that intraspecies divergence in Tartary buckwheat began around 0.42 mya.

**Conclusions:**

The resolution of pan-plastome diversity in Tartary buckwheat provides an important resource for future projects such as marker-assisted breeding and germplasm preservation.

**Supplementary Information:**

The online version contains supplementary material available at 10.1186/s12870-023-04218-7.

## Background

Buckwheat (Polygonaceae: *Fagopyrum*) is a genus of plants that are annuals or perennials, with an herbaceous or semi-shrub habit [1]. Several species in the genus are cultivated for food (primarily the use of seeds as a pseudocereal) and medicine. Foods made from buckwheat are growingly popular because of the presence of balanced essential amino acids, resistance starch, vitamins, minerals and a rich source of bioactive flavonoids, such as rutin, quercetin, (iso) vitexin, and epicatechin [2–4]. The cultivation of buckwheat can be traced back to about 4000 years ago [2, 5]. The *Fagopyrum* genus contains about 15 to 28 species [6] of which only three are cultivated. The cultivated species are *Fagopyrum esculentum* (common buckwheat or sweet buckwheat) which is widely cultivated in Asia, Europe, and the Americas, while *F. tataricum* (Tartary buckwheat) and *F. dibotrys* (golden buckwheat) are mainly cultivated in China [1]. The demand for flour made from *F. esculentum* as a replacement for wheat-based flour is growing given the lack of gluten in the seeds [6, 7]. The seeds of *F. tataricum* are used as an important functional food [6, 8], and *F. dibotrys* is a famous traditional Chinese herbal medicine whose the edible seeds and leaves are eaten to improve health [9–11]. Increasingly, *Fagopyrum* species are being used as an important part in people’s diet, however, characterization of the existing germplasm resources lags behind the need. Assessment of phylogenetic relationships among *Fagopyrum* species is a prerequisite for the initiation of an efficient breeding program [12, 13] in order to predict the viability of crosses and for trait mapping in offspring.

Cultivation of Tartary buckwheat takes place mainly in the high mountain areas of the Himalayan hills and in southwestern China because it is cold-resistant and produces high yields even under poor soil conditions [12, 14]. In addition, the plant is particularly rich in rutin which may have therapeutic potential in treating Alzheimer’s disease [2, 15]. Given the attributes of Tartary buckwheat, horticulturists are ever more interested in breeding new varieties for improved growth and phytochemical composition characteristics for use in larger-scale production. Due to the geographic isolation between populations and localization of artificial selection practiced by traditional farmers, the genetic diversity of Tartary buckwheat germplasm is expected to be quite high [2]. However, given the lack of genomic resources available to study this diversity such tests of genetic diversity are incomplete.

Plastome data has long been used to resolve relationships between plant species because it contains highly conserved genic regions interspersed with more rapidly evolving intergenic regions making comparisons and primers design straight-forward, while also being found in high copy numbers allowing for high yield DNA extraction and deep coverage in genome assembly [16–21]. Additionally, the plastome is uniparentally inherited and nonrecombinant providing a more easily traceable marker in reconstructing phylogenetic relationships [17, 22]. Lastly, the different mutational events in the plastome from loss of entire genes to point mutations can be used to identify clades at varying depths of time based on the frequency of these events occurring [17, 23–27]. But such broad-scale plastome studies have yet to be completed for Tartary buckwheat.

As regards the use of plastomes in studying buckwheats, Cho et al. [28] compared and analyzed the plastomes of two cultivated buckwheats, *F. esculentum*, and *F. tataricum*, and from this inferred the evolutionary relationships between them. Wang et al. [17] analyzed the plastome sequences of *F. esculentum*, *F. tataricum*, *F. dibotrys*, and *F. luojishanense* and resolved the relationships of these four *Fagopyrum* species. Fan et al. [1] compared the plastomes of eight *Fagopyrum* species to determine sequence differentiation, repeat content, and the phylogeny of these species. Further building on previous studies Li et al. [6] analyzed 12 plastomes of *Fagopyrum* and 49 plastomes from other genera in Polygonaceae demonstrating the utility of this type of data in resolving relationships at multiple taxonomic levels. In addition to these studies in *Fagopyrum*, several studies have shown that using a pan-genome approach to analyze plastome DNA sequences can provide useful insights into the origin and diversity of domesticated crops at the population level [29]. For example, Magdy et al. [30] used a pan-plastome developed from 321 complete plastomes to evaluate the genetic variation of cultivated *Capsicum* species leading to an improved classification system between the different cultivars. Wang et al. [31] constructed a pan-plastome using 316 lotus (*Nelumbo* sp.) accessions to investigate the phylogeographic and genetic diversity of the only two extant species in this living fossil lineage. Even though some plastome studies have been conducted for *F. tataricum*, the plastome diversity at the population level has yet to be comprehensively studied.

In this study, we *de novo* assembled 513 complete *Fagopyrum* plastomes (including 506 *F. tataricum* and 7 other *Fagopyrum* species) via the resequencing data of Zhang et al. [2], and combined with 8 complete plastomes of *Fagopyrum* available in NCBI and performed comprehensive comparisons among them. The aim of this work was to: (a) build a reliable pan-plastome for *F. tartaricun*, (b) identify the highly variable regions of plastomes for use in developing new molecular markers, (c) infer population structure and calculate genetic diversity of *Fagopyrum* lineages to provide a basis for wild germplasm resources identification and future cultivar improvement, and (d) reconstruct phylogenetic relationships and divergence times of *Fagopyrum* species to infer the evolutionary history of *Fagopyrum* species.

## Results

### Plastome assembly and analysis of different ***Fagopyrum*** species

Using resequencing data from 510 Tartary buckwheat samples and 7 other *Fagopyrum* samples, a total of 513 complete plastomes were successfully *de novo* assembled and analyzed. The sizes of the completed plastomes ranged from 159,253 bp to 159,576 bp (median = 159,272 bp and ${\bar {\text {x}}}$ = 159,274.35 bp, Table 1). In this study, the newly assembled plastomes all maintained the typical quadripartite structure, consisting of a pair of inverted repeat regions (IRs, IRA and IRB) with sizes ranging from 30,685 bp to 30,852 bp (median = 30,817 bp and ${\bar {\text {x}}}$ = 30,817.04 bp) separated by a large single copy region (LSC) with the sizes from 84,249 bp to 84,875 bp (median = 84,397 bp and ${\bar {\text {x}}}$ = 84,398.65 bp) and a small single copy region (SSC) with sizes from 13,176 bp to 13,414 bp (median = 13,241 bp and ${\bar {\text {x}}}$ = 13,241.61 bp, Table 1). The total GC content of the plastomes ranged from 37.76 to 37.97% (median = ${\bar x}$ = 37.88%), with the IRs ranging from 41.26 to 41.48% (median = ${\bar x}$ = 41.26%), the LSC from 35.96 to 36.29% (median = ${\bar x}$ = 36.20%), and the SSC from 31.97 to 32.98% (median = 32.78% and ${\bar x}$ = 32.77%) (Table 1). Among the four regions, the variation coefficient of size and GC content was the highest in the SSC. In addition, the GC content was higher in the IRs than in the LSC and SSC regions.

**Table 1:** Characteristics of 513 newly assembled *Fagopyrum* plastomes

Length (bp)							GC content (%)					
	Minimum	Maximum	Median	${\bar x}$	σ	CV	Minimum	Maximum	Median	${\bar x}$	σ	CV
LSC	84,249	84,875	84,397	84398.65	24.02	0.028%	35.96	36.29	36.20	36.20	0.022	0.061%
IRA/IRB	30,685	30,852	30,817	30817.04	6.68	0.022%	41.26	41.48	41.26	41.26	0.022	0.054%
SSC	13,176	13,414	13,241	13241.61	10.94	0.083%	31.97	32.98	32.78	32.77	0.072	0.219%
Total	159,253	159,576	159,272	159274.35	23.25	0.015%	37.76	37.97	37.88	37.88	0.011	0.029%

${\bar x}$: Average value. σ: Standard deviation. CV: Variable coefficient.

A total of 77 unique protein-coding genes (PCGs; *rps7, rpl2, ndhB, ycf1, ycf2* each had 2 copies), 30 unique tRNA genes (*trnA-UGC, trnG-UCC, trnI-CAU, trnI-GAU, trnL-CAA, trnN-GUU, trnR-ACG, trnV-GAC* each had 2 copies), and 4 unique rRNA genes (*4.5S rRNA*, *5S rRNA, 16S rRNA, 23S rRNA* each had 2 copies) were identified in the *Fagopyrum* plastomes (Table 2). The distribution of these identified genes in the LSC, SSC, and IRs was as follows: a total of 82 genes in the LSC, including 60 PCGs and 21 tRNA genes (*trnG-UCC* had two copies, only one of which was counted); 11 genes in the SSC, including 10 PCGs and 1 tRNA gene; and 16 genes in the IRs, including 5 PCGs, 7 tRNA genes, and 4 rRNA genes (all genes in the IRs had 2 copies, only one of which was counted). The genes *atpF*, *petB*, *petD*, *rpl16*, *rpoC1*, *rps16*, *trnG-UCC*, *trnK-UUU*, *trnL-UAA*, and *trnV-UAC* in the LSC each contained one intron, while *clpP* and *ycf3* contained two introns. The *ndhA* gene in the SSC contained one intron; *trnA-UGC*, *trnI-GAU*, *ndhB*, and *rpl2* in the IRs each contain one intron (Table 2). The *rps12* gene starts at the middle and upper part of the 5’ end of IRB region, crosses the SSC and IRA regions, and ends at the 3’ end of LSC region, containing an intron (Table 2).

**Table 2:** Annotated genes in *Fagopyrum* plastomes

Gene Category	Functional Group	Gene Name
77 unique protein-coding genes	Subunits of photosystem I	*psaA*, *psaB*, *psaC*, *psaI*, *psaJ*
	Subunits of photosystem II	*psbA*, *psbB*, *psbC*, *psbD*, *psbE*, *psbF*, *psbH*, *psbI*, *psbJ*, *psbK*, *psbM*, *psbT*, *psbZ*
	Small subunit of ribosome	*rps2*, *rps3*, *rps4*, *rps7* (2), *rps8*, *rps11*, rps12*, *rps14*, *rps15*, rps16*, *rps18*, *rps19*
	Large subunit of ribosome	rpl2* (2), *rpl14*, rpl16*, *rpl20*, *rpl22*, *rpl32*, *rpl33*, *rpl36*
	NADH dehydrogenase	ndhA*, ndhB* (2), *ndhC*, *ndhD*, *ndhE*, *ndhF*, *ndhG*, *ndhH*, *ndhI*, *ndhJ*, *ndhK*
	Cytochrome b/f complex	*petA*, petB*, petD*, *petG*, *petL*, *petN*
	Subunits of ATP synthase	*atpA*, *atpB*, *atpE*, atpF*, *atpH*, *atpI*
	RNA polymerase	*rpoA*, *rpoB*, rpoC1*, *rpoC2*
	Large subunit of Rubisco	*rbcL*
	Unknown function	*ycf1* (2), *ycf2* (2), ycf3**, *ycf4*
	Cytochrome c biogenesis protein	*ccsA*
	Envelope membrane protein	*cemA*
	Subunit of ATP-dependent Clp	clpP**
	Translation initiation factor	*infA*
	Subunit of acetyl-CoA	*accD*
	Maturase	*matK*
30 unique tRNA genes	Transfer RNA	trnA-UGC* (2), *trnC-GCA*, *trnD-GUC*, *trnE-UUC*, *trnF-GAA*, trnG-UCC* (2), *trnH-GUG*, *trnI-CAU* (2), trnI-GAU* (2), trnK-UUU*, *trnL-CAA* (2), trnL-UAA*, *trnL-UAG*, *trnM-CAU*, *trnN-GUU* (2), *trnP-UGG*, *trnQ-UUG*, *trnR-ACG* (2), *trnR-UCU*, *trnS-CGA*, *trnS-GCU*, *trnS-GGA*, *trnS-UGA*, *trnT-GGU*, *trnT-UGU*, *trnV-GAC* (2), trnV-UAC*, *trnW-CCA*, *trnY-GUA*, *trnfM-CAU*
4 unique rRNA genes	Ribosomal RNA	*4.5 S rRNA* (2), *5 S rRNA* (2), *16 S rRNA* (2), *23 S rRNA* (2)

* Gene with one intron. ** Gene with two introns. (2) Gene with two copies.

### Analysis of nucleotide variation in ***Fagopyrum*** plastomes

To better understand the nucleotide level differences of all plastomes, 521 complete plastomes comprising of 513 newly assembled *Fagopyrum* plastomes and 8 additional *Fagopyrum* species plastomes were used for the following analysis (Table S1). Compared with the reference genome at a length of 159,272 bp, nucleotide polymorphisms in 7,709 sites were detected among CDSs (coding DNA sequences), exons, introns, and IGSs (intergenic-sequences) of 521 plastomes, which included 5,983 (77.61%) SNVs (single nucleotide variants), 712 (9.24%) mixed polymorphisms (multiple variant types at the same locus in relation to the reference genome, for instance, SNVs and indels at the same locus), 603 (7.82%) InDels (insertions and deletions), and 411 (5.33%) block substitutions (Table S2). We further characterized the distribution of these polymorphisms in the CDSs (2,762 SNVs, 114 mixed polymorphisms, 48 InDels, and 56 block substitutions), exons (33 SNVs, 0 mixed polymorphisms, 3 InDels, and 9 block substitutions), introns (670 SNVs, 95 mixed polymorphisms, 92 InDels, and 44 block substitutions) and IGSs (2,518 SNVs, 503 mixed polymorphisms, 460 InDels, and 302 block substitutions). The results indicated that the total number of polymorphisms was the largest in the IGSs (3,783, 49.07%), followed by the CDSs (2,980, 38.66%), introns (901, 11.69%) and the least in the exons (45, 0.58%) (Table S2).

**Fig. 1:**
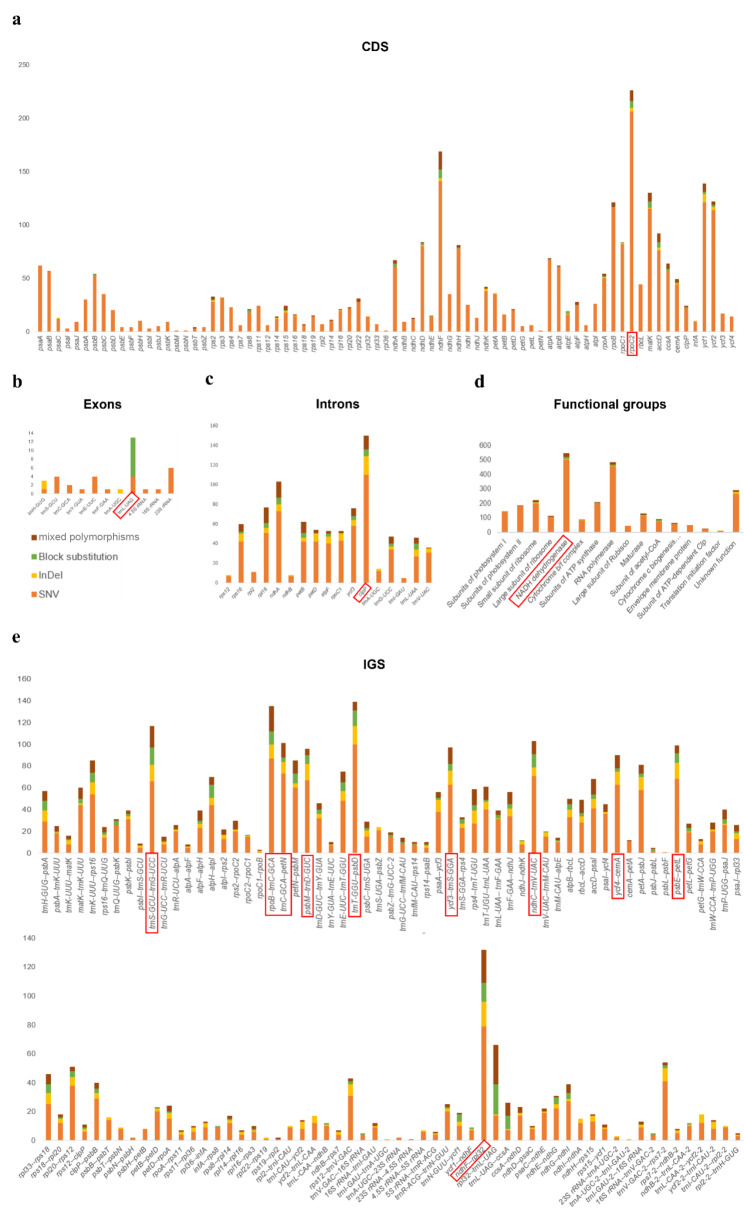
Variant locations categorized by genic position (**a**: CDS, **b**: exons, **c**: introns, and **e**: IGS) and functional grouping (**d**). The red boxes around names indicate the genic position or functional grouping with the highest number of polymorphisms

Among genes, *rpoC2* (in the LSC; 226 polymorphisms) had the most polymorphisms in any CDS region, and *psbM*, *psbN*, *rpl36*, and *petN* had the least, all of which had only one SNV (Fig. 1a). Among exons *trnL-UAG* (in the SSC; 13 polymorphisms) had the most polymorphims, and *trnY-GUA*, *trnF-GAA*, *4.5 S rRNA*, and *16 S rRNA* have the least, all of which had only one SNV (Fig. 1b). Among introns *clpP* (in the LSC; 150 polymorphisms) had the most and the least were in *trnI-GAU* (5 SNVs) (Fig. 1c). The top ten most polymorphic IGS regions were *trnT-GGU–psbD* (LSC; 139 polymorphisms), *rpoB–trnC-GCA* (LSC; 135 polymorphisms), *ndhF–rpl32* (SSC; 132 polymorphisms), *trnS-GCU–trnG-UCC* (LSC; 117 polymorphisms), *ndhC–trnV-UAC* (LSC; 103 polymorphisms), *trnC-GCA–petN* (LSC; 101 polymorphisms), *psbE–petL* (LSC; 99 polymorphisms), *ycf3–trnS-GGA* (LSC; 97 polymorphisms), *psbM–trnD-GUC* (LSC; 96 polymorphisms), *ycf4–cemA* (LSC; 90 polymorphisms) (Fig. 1e). NADH dehydrogenase contained the greatest number of polymorphisms by gene functional group (Fig. 1d). The results of these above assessments of polymorphism indicated that the LSC region is highly variable in *Fagopyrum* plastomes.

### Population structure and genetic diversity

**Fig. 2:**
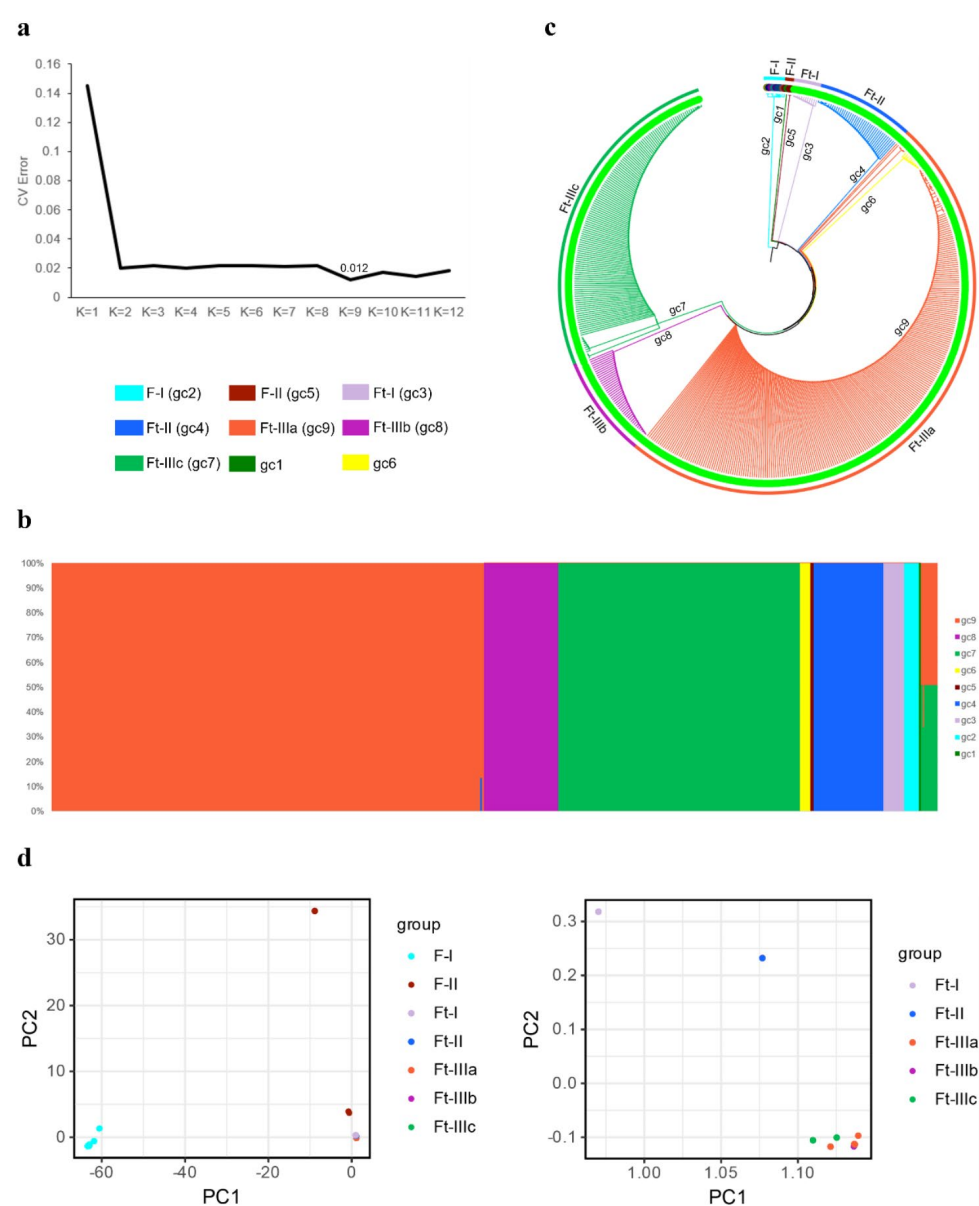
Population structure in *Fagopyrum* pan-plastome. (**a**) CV errors across a range of K values from 1 to 12. The lowest CV error was 0.012 at K = 9. (**b**) Population structure bar-plot at K = 9. (**c**) NJ tree of 521 *Fagopyrum* accessions. (**d**) PCA analysis of 521 *Fagopyrum* accessions and 509 Tartary buckwheat. F-I, F-II, Ft-I, Ft-II, Ft-IIIa, Ft-IIIb, Ft-IIIc, gc-1 to gc-9, are consistently color coded by group in the three figures (b), (c), and (d)

We applied complementary methods to analyze population structure and genetic diversity in order to better understand the matrilineal relationships between *Fagopyrum* species and populations. First, a SNV-only dataset was used to analyze the population structure, the optimal population partitioning was obtained by ADMIXTURE using the minimum CV error which was 0.012 at K = 9 (Fig. 2a). A total of 521 wild and cultivated buckwheat individuals were divided into 9 genetic clusters (gc1 ~ gc9) and 1 mixed genetic cluster (Fig. 2b). Of the genetic clusters 3 of the 9 were composed of individuals of common buckwheat and wild buckwheat with membership including gc1 (*F. esculentum*), gc2 (*F. caudatum, F. qiangcai, F. gracilipedoides, F. rubifolium, F. pugense, F. leptopodum* and *F. luojishanense*) and gc5 (*F. dibotrys*) (Table S1). The other 6 genetic clusters (gc3, gc4, gc6, gc7, gc8 and gc9) and 1 mixed genetic cluster contained different *F. tataricum* individuals (Table S1). The mixed genetic cluster contained *F. tataricum* individuals with nearly 50% probability of being assigned to either gc7 or gc9 (Fig. 2b).

**Fig. 3:**
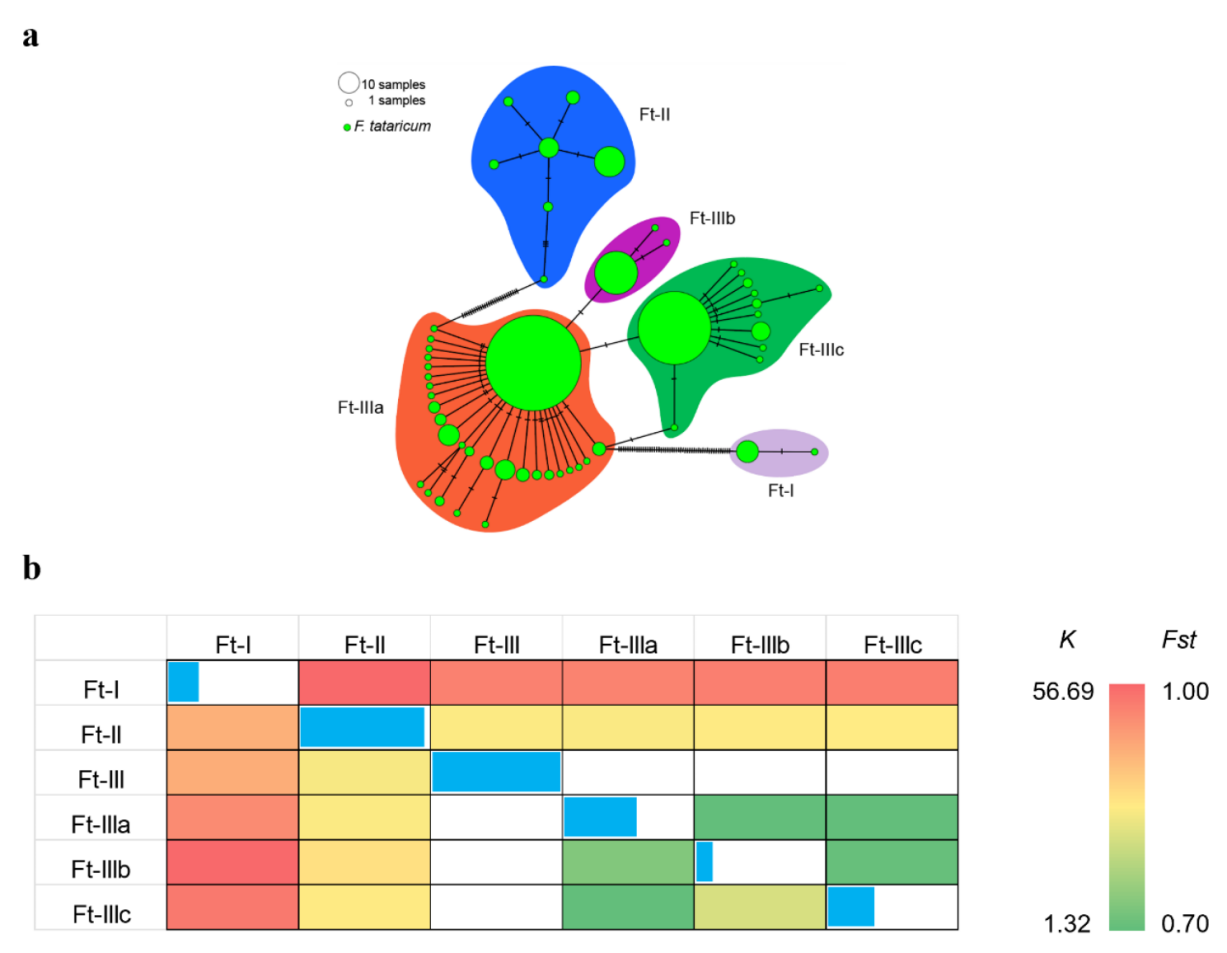
Genetic diversity and population divergence of genetic clusters (Ft-I/II/III, and Ft-IIIa/Ft-IIIb/Ft-IIIc) of Tartary buckwheat. (**a**) Haplotype network of Tartary buckwheat plastomes. (**b**) Genetic distances and population differentiation within and between different genetic clusters. Above diagonal: Pairwise genetic distances (*K*); diagonal: haplotype diversity for each group (the longer the blue bar the greater the Hd); below diagonal: population differentiation coefficient (*Fst*) between different genetic clusters

Then, we further used the SNV dataset to construct a genetic distance tree using a Neighbor-joining (NJ) method (Fig. 2c). Based on this tree and the ADMIXTURE population structure results, common buckwheat and wild buckwheat samples were divided into two genetic clusters, F-I (corresponding to the accessions contained in gc2) and F-II (gc1 and gc5); and the Tartary buckwheat samples divided into five groups and referred to hereafter as Ft-I (gc3), Ft-II (gc4), Ft-III (gc6, gc9, and mixed genetic cluster), Ft-IV (gc8), and Ft-V (gc7). The categories Ft-I, Ft-II, Ft-III, Ft-IV, and Ft-V contained 12, 41, 270, 44, and 142 Tartary buckwheat individuals, respectively. All the Tartary buckwheat individuals clustered together and did not show any evidence of interspecific maternal introgression with other lineages examined in those analyses (Fig. 2c). In other words, modern Tartary buckwheat appears to have originated from a single monophyletic maternal lineage with a selection from multiple sub-lineages thereafter. Principal component analysis (PCA) was conducted to further investigate the genetic diveristy of all 521 individuals based on the SNV-only dataset (Fig. 2d). According to the multi-dimensional analysis based on all buckwheat individuals, the results were consistent with the other analysis and grouped in a similar way, namely, Tartary buckwheat, common buckwheat and all wild buckwheats each resolved in a distinct genetic cluster. The largestest distance in the PCA graph including all individuals showed that the genetic distance between different species groups greatly exceeded that contained within all Tartary buckwheats. The PCA analysis of all Tartary buckwheat individuals resolved three genetic clusters, with the third genetic cluster further subdivided into three groups (Fig. 2d). After analyses with ADMIXTURE, NJ, and PCA, we can reasonably divide all buckwheat accessions into five genetic clusters, namely, F-I, F-II, Ft-I, Ft-II, and Ft-III. All Tartary buckwheat accessions can be divided into three genetic clusters: Ft-I, Ft-II, Ft-III, and Ft-III can be subdivided into Ft-IIIa, Ft-IIIb, and Ft-IIIc. Results from the network analyses (Fig. 3a) also support the three clusters’ arrangement of Tartary buckwheat plastomic diversity but with less support for the subdivision of Ft-III which may be due in part to the stringency with which informative loci are employed. ADMIXTURE, NJ, and PCA all adopt somewhat different algorithms to explain population structure but convergence among them was being employed here to reasonably support the description of genetic diversity found in Tartary buckwheat and nearest relatives.

From 509 Tartary buckwheat plastomes, 53 haplotypes were identified using the SNV-only dataset. According to the haplotype network of plastomes, Tartary buckwheat is clearly divided into three genetic clusters (Ft-I/II/III) and the third genetic cluster can be divided into three groups (Ft-IIIa/Ft-IIIb/Ft-IIIc) albeit with less genetic distance between subgroups (Fig. 3a), which was consistent with the results of the phylogenetic tree and population structure analyses described above. Among the three genetic clusters, the highest haplotype diversity (Hd) was in Ft-III (Hd = 0.71169), followed by Ft-II (Hd = 0.68902), and Ft-I (Hd = 0.16667) (Fig. 3 and Table S3). Among the three groups of Ft-III, the highest Hd was Ft-IIIa (Hd = 0.40419), followed by Ft-IIIc (Hd = 0.25971), and Ft-IIIb (Hd = 0.08985) (Fig. 3 and Table S3). We used *K* and *Fst* values to evaluate the genetic distance and differences between different genetic clusters to assess genetic diversity. As shown in Fig. 3b and Table S4, Ft-I and Ft-II have the largest genetic distance (*K*) between any major genetic cluster, Ft-II and Ft-III have the closest genetic distance between any major genetic cluster. The degree of population differentiation (*Fst*) between Ft-I and Ft-III was the greatest, while the degree of population differentiation between Ft-II and Ft-III was the lowest. Among the genetic subclusters of Ft-III, the genetic distance (*K*) between Ft-IIIb and Ft-IIIc was the largest and between Ft-IIIa and Ft-IIIb the least. When measuring population differentiation with *Fst* the highest value was Ft-IIIb and Ft-IIIc and the lowest was between Ft-IIIa and Ft-IIIc (Fig. 3b and Table S4).

### Divergence time estimation

**Fig. 4:**
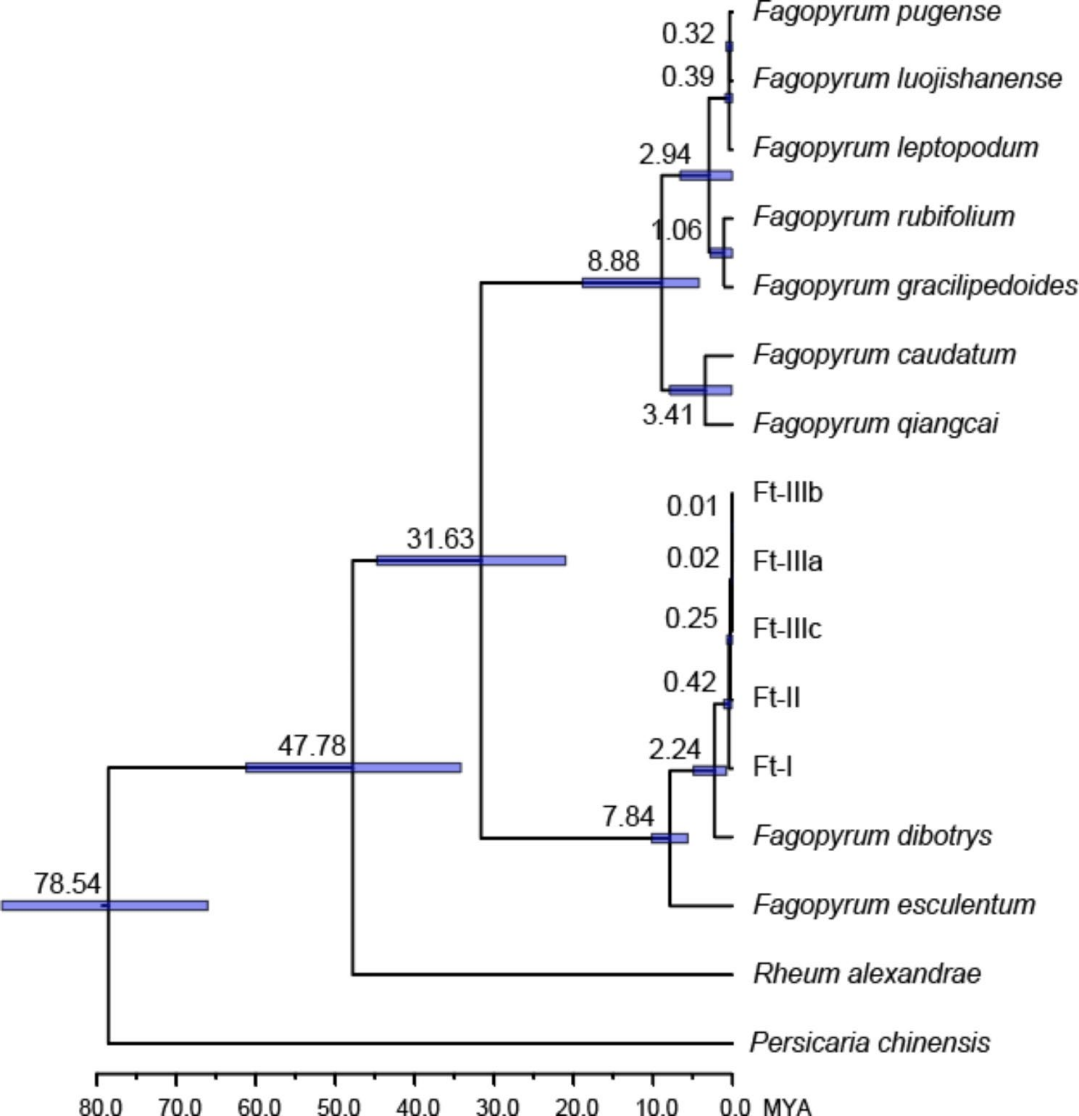
Divergence time estimation based on plastome DNA sequences. Three calibration points were used: the divergence between *Fagopyrum* and *Persicaria* [84.7 mya 95% highest posterior density (HPD): 65.0–95.7], the divergence time between *Fagopyrum* and *Rheum* (44.2 mya HPD: 25.2–55.2), and the divergence time between *Fagopyrum tataricum* and *Fagopyrum esculentum* (7.24 mya HPD: 5.22–9.27)

In order to investigate the phylogenetic divergence time of *Fagopyrum* lineages, we selected representative samples of 10 *Fagopyrum* species (a representative sample was selected from each genetic cluster in *F. tataricum*) and two outgroup species (*Rheum alexandrae* and *Persicaria chinense*, Polygonaceae) for molecular dating (Fig. 4). The results indicated that main split between *Fagopyrum* species occurred about 31.63 million years ago (mya) with the lineage containing *F. pugense*, *F. luojishanense*, *F. leptopodum*, *F. rubifolium, F. gracilipedoides*, *F. caudatum, and F. qiangcai* diversifing about 8.88 mya. The second lineage, which contains *F. tataricum*, *F. dibotrys,* and *F. esculentum* began to diversify around 7.84 mya. Additionally, the crown age of *F. tataricum* was estimated at 0.42 mya, Ft-I, Ft-II, and Ft-III diverged about 0.25 mya and the three groups Ft-IIIa, Ft-IIIb, and Ft-IIIc diverged between approximately 0.01–0.02 mya (Fig. 4).

## Discussion

### Hypervariable regions in the ***Fagopyrum tartaricum*** pan-plastome

In this study, we *de novo* assembled 513 complete *Fagopyrum* plastomes and constructed a pan-plastome of *F. tartaricum* by using 521 *Fagopyrum* plastomes. We found that the plastomes of *Fagopyrum* were highly conserved in quadripartite structure, length, gene order, and GC content, these results are similar to previous studies using smaller samples of *Fagopyrum* species [10, 17, 32]. Compared with the LSC and IRs, the length variation coefficient of the SSC region was the largest, which is likely the result of ongoing contraction and expansion events in the SSC region, indicating that the boundary regions on both sides of SSC are hypervariable regions in which markers could be developed. The GC content of the *Fagopyrum* plastomes were the highest in IR regions, which may be part of the reason why the IR regions are more conserved than the LSC and SSC regions [1, 33, 34].

Notwithstanding structural and genic conservation of plastomes, we still detected numerous nucleotide variations in the *Fagopyrum* pan-plastome. These highly variable sequences could be used for phylogenetic analysis and species identification [17, 35]. Moreover, detecting the highly informative and variable genome regions can be important for diagnostic genetic marker development and DNA barcoding specific to *Fagopyrum* germplasm [6, 36]. In the polymorphism analysis of plastomes of *Fagopyrum*, *rpoC2* had the highest number of polymorphisms of any CDS region, followed by *ndhF*, *ycf1*, and *matK*. The most polymorphic exon region was *trnL-UAG* and intron regions were *clpP* and *ndhA*. The ten most polymorphic IGS regions include *trnT-GGU–psbD*, *rpoB–trnC-GCA*, *ndhF–rpl32*, *trnS-GCU–trnG-UCC*, *ndhC–trnV-UAC*, *trnC-GCA–petN*, *psbE–petL*, *ycf3–trnS-GGA*, *psbM–trnD-GUC*, and *ycf4*-*cemA*. According to the results of these assessments of polymorphic loci, the proportion of variation in the non-coding regions was much higher than that in the coding regions, which is similar to the results reported in *Fagopyrum* previously [1]. This result also supports the numerous findings regarding selection and rate of evolution in non-coding and coding regions, the highly variable non-coding regions are of great value for the study of plant phylogenetics [37–39]. Similarly, the number of polymorphisms in the LSC and SSC regions was higher than that in the IR regions. These results are consistent with the results found in most plant plastomes, which may be related to the relatively higher gene conversion in IRs than in the LSC and SSC regions [1, 33, 35, 40]. These highly variable regions note above, including *trnT-GGU–psbD*, *rpoB–trnC-GCA*, *ndhF–rpl32*, *trnS-GCU–trnG-UCC*, *psbE–petL*, *ycf4*-*cemA*, and *ycf3–trnS-GGA*, have also been detected in previous *Fagopyrum* plastome studies [17]. Previous work on *Fagopyrum* plastomics focused on the variable regions *matK*, *trnL-UAG*, *psbE*-*psbL*, *ndhA, clpP*, and *rpoC2* [6]. Given our results the use of the previously described markers is justified but numerous additional regions can now be considered for studies on the genetic diversity of germplasm resources in buckwheat.

### Phylogenetic relationships and divergence times in ***Fagopyrum***

Complete plastome sequences have been successfully used in phylogenetic studies of many different plant species [17, 41–45]. We reconstructed a well-supported phylogenetic tree based on the complete plastomes of 10 *Fagopyrum* species. The phylogenetic tree supported the monophyly of *F. tartaricum* and resolved a biphyletic pattern for the remaining *Fagopyrum* species. Moreover, our phylogenetic tree indicated that *F. tataricum* was more closely related to *F. dibotrys* than to *F. esculentum*, which is consistent with previous studies [1, 6, 17, 46]. Meanwhile, the close relationship between *F. caudatum* and *F. qiangcai*, as well as between *F. gracilipedoides* and *F. rubifolium* shown in our phylogenetic tree was also reported in previous studies [6, 46]. The relationship between *Fagopyrum* species in our reconstructed phylogenetic tree is consistent with the results of previous studies, which indicates the reliability of whole plastome data in studying plant diversity at multiple taxonomic levels including at the intraspecific level. In addition, it can be inferred from the phylogenetic tree that *F. pugense* is more closely related to *F. leptopodum* than to *F. luojishanense*. This is the first comparative study of the affinities of the three *Fagopyrum* species *F. pugense*, *F. leptopodum*, and *F. luojishanense*. This well-supported phylogenetic tree not only adds to our understanding of the relationships within *Fagopyrum* species (namely *F. tartaricum*), but also provides phylogenetic placement for new species discovered in recent years.

Zhang et al. [2] revealed that Tartary buckwheat originated from Himalayan region and was subsequently domesticated and spread to south China and north China through two geographical routes based on the resequencing data of Tartary buckwheat. Compared with the previous analysis based on the nuclear genome [2], our results enriched the understanding of the genetic structure of Tartary buckwheat population from the perspective of uniparentally inheritance. Similar to the previous results [2], our analysis here supported the three major groups of Tartary buckwheat. In addition, most of these groups contained individuals from three different regions (Himalayan region, south China, and north China) within the same branch, which suggested frequent introgression and hybridization events between different genetic clusters during the domestication of Tartary buckwheat. Our results provided new insights on the evolutionary history of Tartary buckwheat and demonstrated the important value of plastome data in phylogeny and evolutionary biology.

A well-resolved phylogeny, representative taxon sampling, and reliably identified and dated fossils have been shown to be extremely important for the estimation of realistic divergence times [47–49]. Herein we present the first divergence time estimations in *Fagopyrum*. Based on our well-resolved phylogenetic plastome tree and three fossils as temporal constraints, the divergence time of *Fagopyrum* was estimated to be about 31.63 mya, 8.88 mya for wild buckwheat, and 7.84 mya for cultivated buckwheat lineages. The genetic clusters (Ft-I/II/III, and Ft-IIIa/Ft-IIIb/Ft-IIIc) in Tartary buckwheat were also assessed for divergence time and shown to have radiated in the last half million years.

## Conclusions

We used 521 plastomes composed of 513 newly assembled plastomes and 8 downloaded from NCBI to construct the pan-plastome of *F. tartaricum*. Although the plastome is highly conserved in terms of structure, composition, and gene content, there are still abundant polymorphisms from which to resolve maternal lineage diversity. In this study, we systematically analyzed the nucleotide variability of the *F. tartaricum* pan-plastome. From this we found that in addition to the highly variable regions reported in previous studies, several new regions with high levels of variability could be employed as new molecular markers for phylogenetic and species identification studies. Based on the SNV dataset of the pan-plastome and using several different population genetic structure analysis methods, we divided these *Fagopyrum* species into five genetic clusters (F-I/II, Ft-I/II/III). By analyzing the phylogenetic relationship of *Fagopyrum* species, we found that all Tartary buckwheat individuals clustered together forming a monophyletic lineage. So, while the domestication of Tartary buckwheat appears to have originated from several intraspecific genetic clusters, no evidence of interspecific maternal introgression was found in our dataset. That said the presence of 10 mixed lineage plastomes suggests that these individuals may be the result of intraspecies maternal introgression resulting in heteroplasmic offspring or relict intermediates. More work is needed to substantiate these hypotheses including comparisons to the nuclear genome and assessments of the phenotype for these individuals. Such individuals of hybrid origin could be of great value in producing elite cultivars if heterosis is present among such individuals.

The haplotypes of Tartary buckwheat were divided into three primary genetic clusters (Ft-I/II/III). The haplotype diversity index of each genetic cluster, the genetic distance between different genetic clusters and the population differentiation index were analyzed to understand the genetic diversity of Tartary buckwheat populations, which will be essential in future breeding and improvement programs. In this study, the divergence time of each species in *Fagopyrum* was evaluated for the first time. In addition, the divergence time of each genetic cluster within Tartary buckwheat population was also evaluated. As such this is the first systematic analysis of the divergence history of *Fagopyrum* species, giving us a better understanding of the history of *Fagopyrum* species.

## Methods

### Plant materials

A total of 521 complete *Fagopyrum* plastomic data were available in this study, of which 513 were resequenced from Zhang et al. [2], including 506 *F. tataricum* and 7 other *Fagopyrum* species, which we downloaded from the ENA database (https://www.ebi.ac.uk/ena/browser/; Study accession No. PRJNA600676) and *de novo* assembled to obtain 513 complete *Fagopyrum* plastomes (the assembly method is described in the next section), and the remaining 8 complete *Fagopyrum* plastomes were downloaded from the NCBI database (Table S1).

### Sampling, assembly, and annotation

Illumina paired-end reads from whole-genome sequencing (WGS) of wild and cultivated *Fagopyrum* species were downloaded from the ENA database (https://www.ebi.ac.uk/ena/browser/) [2]. The clean raw WGS reads were aligned against published *Fagopyrum* plastomes to extract out plastid-origin reads by using bwa v0.7.17 [50] and SAMtools v1.9 [51]. The plastid-origin reads were then used for *de novo* assembly of complete plastomes by using SPAdes v3.15.2 [52] and Bandage v0.8.1 [53] following the pipelines described in He et al. [29]. The orientations of LSC and SSC in all the obtained plastome sequences were manually adjusted to maintain maximum collinearity with each other in MEGA7 [54]. The assembly metrics of plastomes, e.g., total length and GC content of different structures including LSC, SSC, and IR regions was calculated by using a customized Perl script and were further analyzed in IBM SPSS Statistics 22 (SPSS Inc., Chicago, USA). Gene annotation of the plastomes was performed using Geseq online tools [55] and was manually checked and modified if necessary.

### Population structure

The assembled complete plastome sequences were aligned in MAFFT 7 [56] with optimal settings determined by the program (“—auto” option). The obtained alignment was carefully checked and adjusted manually for each mismatch and gap locus to generate a corrected alignment, which was then analyzed by DnaSP 6 [57] for calling sequence variations between different plastomes. A previously published plastome (*Fagopyrum tataricum*; GenBank: MT712164.1) was used as a reference genome for this analysis. Functional annotation of all nucleotide variations was performed with SnpEff software [58]. Different variations located in different plastomic regions were summarized and visualized in Microsoft Excel 2019 (Microsoft corporation, USA). Population structure of all *Fagopyrum* accessions was inferred based on plastomic SNVs using ADMIXTURE 1.3 [59] with default settings, with K running from 1 to 12. The ADMIXTURE results were assessed by the lowest CV error which was used to select the optimal K to assign accessions with a membership probability (Q value) ≥ 0.65 to the corresponding genetic cluster. Accessions with all membership probabilities < 0.65 were assigned to a mixed group. A Neighbor-joining tree based on p-distance calculation was generated by using plastomic SNVs in MEGA7 [54].

### Haplotype and genetic diversity analyses

The haplotypes of plastomes were determined in DNAsp 6 [57] with the option of excluding gaps and missing loci based on the corrected alignment of complete plastome sequences. Haplotype networks were inferred and plotted in Popart v1.7 [60] with a median-joining method to investigate the genealogical relationships of the identified haplotypes. The output figure was further modified to be more readable in Adobe Illustrator software (Adobe Systems Incorporated, USA). The programs DNAsp 6 [57] and MEGA7 [54] were used to calculate the haplotype diversity for each group of haplotypes, and the evolutionary distances based on the Tajima-Nei distance model, and population differentiation index (*Fst*) between different groups by using the plastomic SNVs. The principal coordinates analysis (PCA) was conducted in TASSEL 5.0 [61].

### Phylogenetic analysis and molecular dating

To perform the phylogenetic analysis, complete sequences of plastid genomes were aligned in MAFFT 7 [56] and manually adjusted in MEGA7 [54]. A Neighbor-joining tree was generated by using plastomic SNVs in MEGA7 [54]. For the molecular dating, a set of representative accessions from each species and the major genetic clusters (Ft-I/II/III and subclusters from Ft-III) were selected for this analysis. A relaxed lognormal molecular clock with Yule priors was chosen to estimate the divergence time of lineages as implemented in BEAST v1.8.4 [62]. Three calibration points, from the TimeTree website (http://www.timetree.org/) were employed: the divergence between *Fagopyrum* and *Persicaria* [84.7 Mya 95% highest posterior density (HPD): 65.0–95.7], the divergence time between *Fagopyrum* and *Rheum* (44.2 Mya HPD: 25.2–55.2), and the divergence time between *Fagopyrum tataricum* and *Fagopyrum esculentum* (7.24 Mya HPD: 5.22–9.27). Following the suggestion of Ho (2007), we assigned a normally distributed prior for the three calibrations, in which standard deviations of 6.7, 6.7, and 1.2 were used, respectively.

## Electronic supplementary material

Below is the link to the electronic supplementary material.

Supplementary Material 1

## Data Availability

The datasets generated and analysed during the current study are available in the Figshare repository, (https://figshare.com/) with DOI: 10.6084/m9.figshare.21731153. All other data and material analyzed in the current study are included in the manuscript and the supplementary information.
